# Adamantinoma-Like Ewing Sarcoma of the Parotid Gland: A Systematic Review

**DOI:** 10.7759/cureus.80477

**Published:** 2025-03-12

**Authors:** Theoklitos Tsaprazlis, Konstantinos E Stavrakakis, Foivos I Kaldis, Georgios Kostakis, Dimitrios Filippou

**Affiliations:** 1 School of Dentistry, National and Kapodistrian University of Athens, Athens, GRC; 2 Evgenidio Hospital, National and Kapodistrian University of Athens, Athens, GRC; 3 Department of Anatomy, National and Kapodistrian University of Athens, Athens, GRC

**Keywords:** adamantinoma-like, ewing, parotid gland, salivary gland, sarcoma

## Abstract

Adamantinoma-like Ewing sarcoma (ALES) is a rare malignant neoplasm, identified as a type of Ewing sarcoma (ES), characterized by the EWSR1::FLI1 translocation and a complex immunoprofile. Its various clinical, histopathological, and immunohistochemical features, along with a multitude of diseases in the differential diagnosis, make it a complex entity that requires special attention. Treatment involves parotidectomy alone or in combination with adjuvant chemoradiotherapy.

A systematic electronic search was conducted through February 2025 in the PubMed and Scopus databases utilizing relevant Medical Subject Headings, without any time constraints. A total of 35 publications were identified using the keywords "adamantinoma-like Ewing sarcoma" AND "parotid". Following the Preferred Reporting Items for Systematic Reviews and Meta-Analyses (PRISMA) guidelines, a total of 15 articles were included in this systematic review, all of which were case reports.

Based on the limited information available, it appears that this tumor frequently demonstrates regression after treatment. However, given the small number of recorded cases, a clear survival rate associated with specific therapies remains uncertain. This highlights the importance of ongoing assessments and follow-ups to establish definitive outcomes for patients.

## Introduction and background

Mesenchymal tumors are infrequent in the salivary glands, with sarcomas representing merely 0.3-1.5% of malignant salivary gland tumors [1,2]. Ewing sarcoma (ES) is a rare bone and soft tissue sarcoma primarily seen in younger individuals, marked by sheets or clusters of small, uniform round blue cells characterized by vesicular nuclei and scant, relatively transparent cytoplasm [3,4]. Adamantinoma-like Ewing sarcoma (ALES) is an uncommon tumor now recognized as a variant of ES due to its similar round cell structure, CD99 (MIC2) and NKX2.2 immunoreactivity, along with its complex epithelial differentiation, defined by the t(11;22) EWSR1::FLI1 gene fusion [5-8]. Initially reported by Bridge in 1999 as arising in the long bones, ALES is now recognized to mainly occur in the head and neck region, primarily in the salivary glands, as well as in the sinonasal region, the thyroid gland, and the eye socket [9,10]. ALES in the salivary glands presents a considerable risk for misdiagnosis because of its basaloid morphology and the presence of both epithelial and mesenchymal markers, resembling various basaloid neoplasms [10]. Moreover, the varied traits of ALES, its common clinical sites, the resemblance in immunohistochemical markers, and the overlapping histological and molecular attributes position it among the differential diagnoses of several neoplasms [10].

This systematic review aims to gather and critically analyze findings from existing literature on this rare tumor variant of the parotid gland, concentrating on its clinical, molecular, and histopathological characteristics while also exploring differential diagnoses and treatment strategies for this sarcoma.

## Review

Materials and methods

This systematic review was conducted in accordance with the PRISMA (Preferred Reporting Items for Systematic Reviews) guidelines, which provide a framework for performing systematic reviews. To facilitate our study, a systematic electronic search was performed in the PubMed and Scopus databases using the search terms "adamantinoma-like Ewing sarcoma" AND "parotid", without applying any filters. Due to the numerous cases documented across various body organs, the authors focused solely on relevant articles detailing diagnosed cases of ALES in the parotid gland. Exclusion criteria for the systematic review comprised studies unrelated to this subject, articles published in languages other than English or Greek, and duplicate publications. Each of the authors participated in selecting the studies, extracting data, and evaluating the quality of the review.

Results

**Figure 1:**
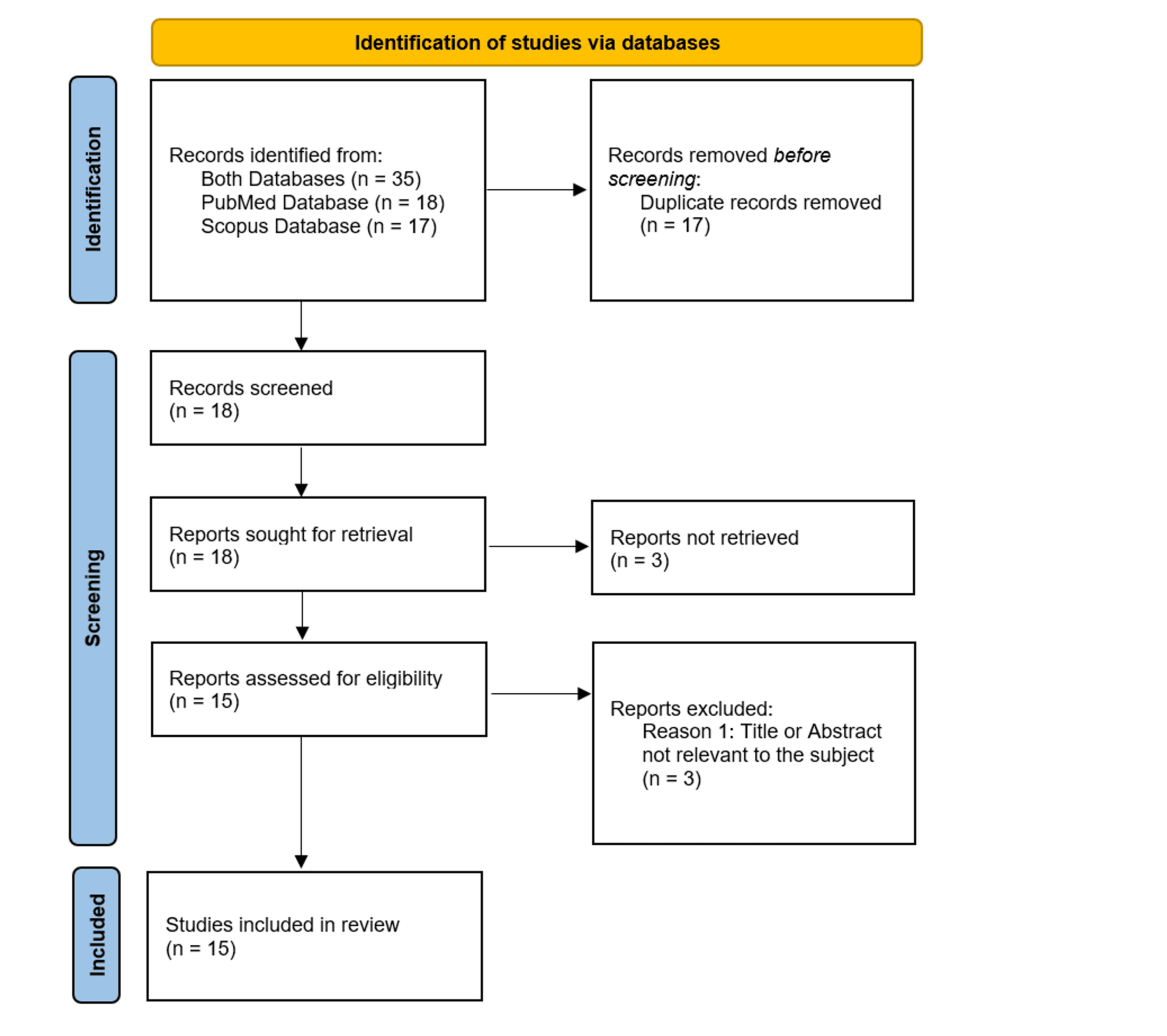
PRISMA flow diagram of the included studies. PRISMA, Preferred Reporting Items for Systematic Reviews.

The preliminary search returned 35 articles from the PubMed and Scopus databases, with no restrictions on the year of publication. Twenty articles were excluded based on defined criteria; among them, three were unrelated to the systematic review's topic, while the remaining 17 were duplicates. A secondary manual literature search of the selected articles yielded no additional entries. Consequently, all 15 articles included in this systematic review were case reports. The search approach for the specified databases is illustrated in the PRISMA flow diagram in Figure 1.

Discussion

Demographic Characteristics

The analytical systematic review of 15 articles included an assessment of 27 reported cases of ALES located in the parotid gland. Among these patients, 16 were female [1-2,4-8,11-15] and 11 were male [3-5,8-10]. Most patients were adults, comprising 25 individuals [1,3-15], while two were minors [2,8]. Their ages ranged from 12 to 79 years, with an average age of 46.73 years [1-15].

Radiological and Clinical Findings

The findings suggest that ALES may clinically manifest as a mass or swelling in the facial [5,11,13], neck [1,5,13], parotid [3-6,10,12], or preauricular [2,4,6,15] regions. Most patients with ALES presented with painless symptoms [1,5,6,8,10,11,13], though some tumors were painful or tender [2,3]. Clinical examination revealed tumor sizes ranging from 2 to 7.9 cm [2,3,5,6,8,10,13-15], while imaging studies reported masses reaching up to 8.8 cm [4]. Additionally, five patients underwent CT scans, which revealed a solid, ill-defined, or heterogeneous mass of various dimensions ranging from 1.5 to 8 cm [1,4,6,12,15]. Two cases had MRI, showing a solid, homogeneous mass [10] alongside an oval enhancing lesion [15], which affected both the superficial [10,15] and deep lobes [10] of the parotid gland, extending into the lateral cortex of the left mandibular ramus [15]. Lastly, one patient underwent an ultrasound, which revealed a solid mass [15] alongside clinical evaluation.

Histological Features

Microscopic evaluation is essential for confirming a diagnosis and provides valuable insights into ALES cases. The article analysis indicated that tumors resided within the salivary gland parenchyma [4,5,13] and were surrounded by hypercellular [2,3,14,15], desmoplastic, or fibrotic [1,3-5,8-11,12,14,15] stroma. This stroma exhibited scant metachromatic [3] characteristics, with mild to moderate lymphoplasmacytic infiltration and scattered histiocyte clusters [15]. Architectural patterns varied among tumors, displaying prominent nests [1-6,8-11-13,15], cords [1,6,9,12,15], sheets [2,4,8,9,12,13], islands [9,10,14], and clusters [2,3,10,14] of epithelioid cells with relatively bland nuclei [3], demonstrating peripheral palisading [1,3,8-10], trabecular formations [5,10], lobules [4,5,10,11], and follicle-like spaces [8,10]. Rare instances included nuclear molding [10,14], and some cells exhibited cytoplasmic vacuolation [10], with a tigroid background noted near certain clusters [10]. Well-defined rosettes were identified in some cases [1,2,4,5,10].

Additional observations included keratinization [8,9,13], hyalinization [10], central cystic degeneration [10], squamous differentiation [3,4,8,10,13], chondro-osseous differentiation [4], calcification [8], and peritheliomatous patterns [2]. The tumor cells appeared small [2-4,6,10,12-14], with shapes ranging from round [2-4,6,8,12-14] to ovoid [2,9] and blue [2-4,12,13], characterized by monotonous/monomorphic [3,5,6,8,10,12,14] features. Their cytoplasm exhibited minimal variation, with regions appearing clear to basophilic [5], eosinophilic [1,4,9], amphophilic [3], or indistinct [3,4,10,12], alongside focal cytoplasmic vacuolization [3].

Nuclear traits included round [5,10,13], vesicular [3,5], large [10], angulated [14], or monomorphic [4,6,10,15] forms, featuring fine [1,2,4,10,12,13], vesicular/granular [1,2,4,5,12], or evenly dispersed [10] chromatin with a prominent nucleolus [2,4,5,12,15]. In two instances, a high N:C ratio was reported [9,14]. One case noted loose clusters of epithelioid cells intermixed with lymphocytes [3], while another displayed a salt-and-pepper chromatin pattern indicative of neuroendocrine features [4]. However, basaloid morphology was observed in some tumor cells [2-4,8,11,14,15].

The tumor cells extensively infiltrated surrounding gland acini and fibroadipose tissues [5,10], with duct entrapment noted in various instances [1,2,4,10,12]. Tumor margins were recorded as either positive [4,8] or negative [6,8]. Some reports indicated local expansion into perisalivary soft tissues, illustrating vascular [2,4,6,8], lymphovascular [4], and perineural invasion [4,6,8,10], as well as infiltration of skeletal muscle structures [6,10]. Increased mitotic activity [2-5,8,11,12] and apoptotic cells [9] were observed, along with widespread necrosis [4,5,8,11,12,14].

Diagnosis

Diagnosing ALES poses challenges due to the various markers it may express. The immunohistochemical review indicated that all patients were positive for the CD99 marker [1-13,15], except for one patient reported by Wakely et al. [14], where no relevant immunohistochemical marker contributed to the diagnosis, highlighting a misinterpretation of a fine needle aspiration (FNA) followed by uninformative flow cytometry. Other immunohistochemical positives included NKX2.2 [3,5,8,10,13,15], keratin AE1/AE3 [1,3,5,7,8,12,15], keratin 19 [15], p40 [1,3,5,7-9,11-13], p16 [1,4], p63 [2,4,7,9,10,12,15], synaptophysin [3-6,12,13], PanCK1 [1,11], β-catenin [1], CD56 [6], CD117 [10], CK8/18 [4,6], CK5/6 [4], GATA3 [15], INI1 [15], EMA [8], Fli-1 [2,8], pan-cytokeratin [7,10,12], cytokeratin [2], keratin 20 [12], CAM5.2 [7], SYN [7], CHR [7], and PGP9.5 [4]. Certain patients were found to be focally positive (<5% of cells) for markers including synaptophysin [1,4,5,8], chromogranin [1,4,5], S100 [2,5], SYN2 [1,11], cytokeratin 7 [1], cytokeratin 20 [1], desmin [1], α-SMA [1], TTF-1 [1], LEF-1 [1], DOG1 [1], androgen receptor [1], CHR3 [1], actin [1], NUT-1 [1], p63 [6], p40 [6], p16 [4], CK5 [7], CK20 [4], and CD56 [3,4]. The Ki-67 index was documented in four articles, ranging from 10% to 60% [4,6,8,15].

Analysis from the articles indicated that patients were negative for S100 [1,3,5-9,11,12,15], SOX10 [7,8,12], synaptophysin [5,8,10,15], chromogranin [3-5,8,10,13,15], actin [5,11], desmin [4,5,8,11,12,15], NUT-1 [5,6,8,10,11], in situ hybridization for HPV DNA [1], PAS [1], CK7 [4,6,10], CK20 [3], TTF-1 [3,4,6,12], androgen receptor [6], keratin 7 [12,15], mammaglobin [15], ER [15], β-catenin [8], p16 [8], SMARCB1 [8], CHR3 [11], CD3 [12], CD45 [3], CD56 [4,7], CD117 [4], smooth muscle myosin heavy chain [3,12], smooth muscle actin [2,10,12], calponin [12,15], CDX2 [4], GFAP [4], vimentin [4], HER2 [4], alpha-fetoprotein [2], HepPar-1 [2], INSM1 [10], DOG1 [10], NR4A3 [10], MYB [10], ER [12], PR [12], GATA3 [12], LCA [12], PAX5 [12], and SV40 [12].

FISH testing for EWSR1 and FLI1 gene rearrangements yielded positive results in almost every recorded case, indicating a translocation [1-6,8-13,15], with one case showing negativity [7]. In some instances, fine needle aspiration (FNA) contributed to the diagnostic process [1,3,12,14], supplemented by flow cytometry [14], which yielded no useful results. As emphasized earlier, diagnosing ALES can be a multifaceted challenge that may lead to misdiagnoses. This occurs due to the diverse characteristics of ALES, its frequent clinical localization, the similarity of its immunohistochemistry markers, and its overlapping histological and molecular features with other tumors. Therefore, it is crucial for clinicians to comprehend all potential differential diagnoses related to this condition. A list of neoplasms included in the differential diagnoses for ALES is provided in Table 1.

**Table 1:** The neoplasms included in the differential diagnosis for ALES with the associated bibliographic references. ALES: adamantinoma-like Ewing sarcoma.

Neoplasms	References
Basal cell carcinoma	[1,2,4-8,10-13,15]
Neuroendocrine carcinoma	[1,4-8,10,12]
Adenoid cystic carcinoma	[1,10-13,15]
Sialoblastoma	[2,6,8,10]
Basal cell adenoma	[1,5,6,8]
Ewing sarcoma	[2,10,12,15]
Merkel cell carcinoma	[5,12,13]
Adenocarcinoma	[4,6]
Myoepithelial carcinoma	[10,15]
Salivary gland neoplasms	[3,10]
Lymphoproliferative disorder	[3,10]
Metastatic carcinoma	[3,10]
SMARCB1-deficient carcinoma	[15]
Desmoplastic small round cell tumor	[11]
Synovial sarcoma	[11]
Epithelioid neoplasms	[3]
Pleomorphic adenoma	[2]
Primitive neuroectodermal tumor	[2]
Acinic cell carcinoma	[10]
Any malignant small round cell tumor with basaloid cells	[14]
Hyalinizing clear cell carcinoma	[1]

Treatment

The analysis of treatment case reports for ALES indicated that parotidectomy was the predominant surgical approach [1-13,15]. Specifically, five patients underwent radical parotidectomy [1,4,6,10], while four patients had superficial parotidectomy [2,3,12,15] along with facial nerve dissection. Chemotherapy was administered to 14 patients [1,4-6,8,9,13,15], and radiotherapy was given to 13 patients [1,4-6,8,9,13,15]. Additionally, modified radical neck dissection was performed in three cases [4,15], while cervical lymph node excision was carried out in one case [2].

Prognosis and Follow-up

Among the reported cases of patients undergoing treatment for ALES, only 18 provided details regarding prognosis and follow-up [4,5,7-9,13,15]. The follow-up period varied, with some cases extending up to 96 months post-treatment [13]. Of the patients monitored for follow-up, 14 exhibited no signs of recurrence, disease, or metastasis [4,5,8,9,13,15], three were alive with the disease [4,5,7], and one patient succumbed to the illness [5].

## Conclusions

In summary, ALES is a rare entity that has recently been identified in the head and neck region, particularly in the parotid gland. It is marked by a diverse array of histopathological characteristics and predominantly affects adults. The clinical presentation of this neoplasm can vary, as can the conditions that must be considered in its differential diagnosis. Therefore, cytopathologists should remain vigilant and include this tumor in a broad differential diagnosis due to its unique histopathological and immunohistochemical features, especially given the limited references available in existing literature. From the scant data available, it appears that this neoplasm often shows regression following therapeutic interventions, whether through parotidectomy alone or in combination with adjuvant chemoradiotherapy. Nevertheless, due to the small number of documented cases, a definitive survival rate associated with specific treatments remains unclear, making regular evaluations and follow-ups essential for establishing more reliable outcomes.
